# Disease insights through cross-species phenotype comparisons

**DOI:** 10.1007/s00335-015-9577-8

**Published:** 2015-06-20

**Authors:** Melissa A. Haendel, Nicole Vasilevsky, Matthew Brush, Harry S. Hochheiser, Julius Jacobsen, Anika Oellrich, Christopher J. Mungall, Nicole Washington, Sebastian Köhler, Suzanna E. Lewis, Peter N. Robinson, Damian Smedley

**Affiliations:** University Library and Department of Medical Informatics and Epidemiology, Oregon Health & Science University, Portland, OR USA; Department of Biomedical Informatics and Intelligent Systems Program, University of Pittsburgh, Pittsburgh, PA 15206 USA; Skarnes Faculty Group, Wellcome Trust Sanger Institute, Wellcome Trust Genome Campus, Hinxton, Cambridge, CB10 1SA UK; Genomics Division, Lawrence Berkeley National Laboratory, 1 Cyclotron Road, Berkeley, CA 94720 USA; Computational Biology Group, Institute for Medical Genetics and Human Genetics, Universitatsklinikum Charité, Augustenburger Platz 1, 13353 Berlin, Germany

## Abstract

New sequencing technologies have ushered in a new era for diagnosis and discovery of new causative mutations for rare diseases. However, the sheer numbers of candidate variants that require interpretation in an exome or genomic analysis are still a challenging prospect. A powerful approach is the comparison of the patient’s set of phenotypes (phenotypic profile) to known phenotypic profiles caused by mutations in orthologous genes associated with these variants. The most abundant source of relevant data for this task is available through the efforts of the Mouse Genome Informatics group and the International Mouse Phenotyping Consortium. In this review, we highlight the challenges in comparing human clinical phenotypes with mouse phenotypes and some of the solutions that have been developed by members of the Monarch Initiative. These tools allow the identification of mouse models for known disease-gene associations that may otherwise have been overlooked as well as candidate genes may be prioritized for novel associations. The culmination of these efforts is the Exomiser software package that allows clinical researchers to analyse patient exomes in the context of variant frequency and predicted pathogenicity as well the phenotypic similarity of the patient to any given candidate orthologous gene.

## Introduction

Despite the many recent successes in identifying causative mutations for human heritable diseases through the use of new sequencing technologies, an associated gene has not been identified for approximately half of the ~7000 diseases (Boycott et al. 2013) with current progress at 150–200 new disease-gene identifications per year (http://www.irdirc.org). Discovery of these genotype-to-phenotype relationships is the critical first step towards understanding the mechanism of these heritable diseases and developing potential new treatments.

Although new technologies such as whole exome sequencing (WES) are cost effective and fast, they typically generate thousands of potential candidate variations that need to be interpreted in light of what is known or can be predicted about the variant and the affected gene. One of the most powerful lines of evidence comes from whether the patient’s clinical signs and symptoms show similarity to phenotype data previously associated with mutations in the gene.

A wealth of data for this task is available in the Mouse Genome Database (MGD) (Eppig et al. 2015) through the curation efforts of the Mouse Genome Informatics (MGI) group and from the high throughput phenotyping of the International Mouse Phenotyping Consortium (IMPC) (Koscielny et al. 2014). The paper by Meehan et al. in this issue describes how IMPC aims to complete the functional catalogue of all protein-coding genes by 2020, strengthening the existing status of the mouse as the premier model organism for investigating human disease.

The MGI and IMPC website resources are available to clinical researchers to assess individual human disease variant candidates. However, until recently this data have been under-utilized and not used in an automated, systematic approach due to the challenges in comparing human and mouse phenotypes and the lack of tools allowing clinicians and researchers to perform these comparisons (Gkoutos et al. 2012). In this review, we discuss the challenges in comparing phenotypes across species and integration with exome analysis, some of the solutions that have been developed in the context of the Monarch Initiative (www.monarchinitiative.org), and emerging tools for rare disease exome analysis that exploit these comparisons.

## Clinical and model organism phenotype data

Data on the ~7000 known genetic and other rare human diseases are stored in the Online Inheritance in Man (OMIM) (Amberger et al. 2015). OMIM contains substantial amounts of descriptive data on the objective signs and subjective symptoms for each disease. However, as this data are represented as free text, it is less amenable to computational analysis, e.g. related diseases cannot easily be discovered using these descriptions. The Human Phenotype Ontology (HP) was developed to describe such phenotypes in a standardized manner that allows such analyses (Köhler et al. 2014a) and there are now over 11,000 terms in HP. The results of an ongoing curation effort by the Monarch Initiative, and members of the rare disease community such as Orphanet (Ayme 2003), are made publicly available from http://www.human-phenotype-ontology.org and currently contain annotations for 9019 DECIPHER, OMIM, and Orphanet disorders.

The largest source of mouse phenotype data is the MGD, containing curated annotation of mouse mutants described in literature and also by the import of large-scale projects such as IMPC. Phenotypes are described using the well-established Mammalian Phenotype Ontology (MP) developed precisely for this curation effort. MP currently contains 10,000 terms (Smith and Eppig 2012). MGD contains 278,701 phenotype annotations for over 53,000 different mouse strains involving disruptions in 10,753 genes. The IMPC database contains data for 1470 strains, each with a presumptive null mutation in a unique gene, and 5725 phenotype annotations. The IMPC pipeline involves a sequential set of tests collecting data on parameters covering all major adult organs and most major disease areas (Koscielny et al. 2014). Given the focussed nature of most published studies, phenotypes that are not assigned to a MGD strain cannot be assumed to be absent. In contrast, for the standardized IMPC pipeline, every assayed phenotype can be assumed to be negative if not reported. However, the pipeline only covers a defined but limited range of phenotypes.

At present some 3400 human genes have HP annotations assigned to them based on their association with disease(s). Mouse mutants involves only a single gene disruption and MP annotation(s) exist for 9974 genes, with only 2341 overlapping with the set of human disease genes. Therefore there is an abundance of genes with genotype–phenotype information available only in the mouse and potentially translatable to human disease studies.

The Monarch Initiative (www.monarchinitiative.org) is an international consortium that aims to integrate data from a large number of diverse resources for human and model organisms (including from IMPC, MGD, OMIM, Orphanet, etc.) describing diseases, phenotypes, environmental factors, drugs, literature, research resources, etc. for the purposes of disease mechanism discovery and diagnosis. The foundation of the Monarch Initiative is the semantic integration of genotype–phenotype data into a single knowledge base that provisions for the application of graph-based computational analyses through the OWLSim software package, including phenotypic profile matching (Washington et al. 2009). Flexible tools for data access and retrieval through APIs and Web widgets suitable for inclusion in third-party sites support the customization and use of this data for diverse purposes.

## Cross-species phenotype mapping

The biggest barrier to computational use of the mouse genotype–phenotype associations for human disease research is the use of different phenotype ontologies by the two communities. For example a computer, or even a non-specialist researcher, would not know that the HP term *craniosynostosis* (HP:0001363) is equivalent to the MP term *premature suture closure* (MP:0000081). Mungall et al. 2010 described a process called “logical decomposition” that could be used to define the species-specific phenotype terms using generic, species-agnostic ontologies to computationally define the terms in the species-specific ontologies. Each term is broken down to a combination of a quality (Q), representing what is abnormal about the entity, and an entity (E), representing the anatomical structure or biological process (Köhler et al. 2013; Washington et al. 2009). The entity terms come from well-established ontologies such as the Gene Ontology (GO 2015), the Chemical Entities of Biological Interest [CHEBI; (Hastings et al. 2013)] ontology, or the UBERON multi-species anatomy ontology (Mungall et al. 2012; Haendel et al. 2014). The Phenotype and Trait Ontology (PATO) is used for the qualities. In the above example, both the HP and MP terms are represented by the *premature closure* (PATO:0002166) of the *suture* (UBERON:0000969) and therefore can be detected as equivalent by an algorithm. In this manner, the logic underlying HP and MP is being co-developed by members of the Monarch Initiative and MGI.

This approach has been applied to human disease, mouse, and zebrafish datasets. Known disease genes were detected with high specificity and sensitivity by semantic phenotype comparisons (Köhler et al. 2013; Washington et al. 2009). The algorithm performs pairwise comparisons between each disease and animal phenotype. Related but non-exact matches can be detected by taking advantage of the hierarchical structure of the ontologies; e.g. a clinical phenotype of *speech articulation problems* and a mouse mutant exhibiting abnormal *larynx morphology* would share a common phenotype of *abnormality of the larynx*. Each match is scored using measures of semantic similarity (Pesquita et al. 2009) such as the Jaccard index or the Information Content of the common phenotype match. The similarity between the disease and animal model is then given by an aggregated score between all the matches, such as the average score across all possible matches or the score of the best pairwise match.

## Tools for exploring mouse models of human disease

A number of resources have taken advantage of the cross-species phenotype matching approach to develop websites to generate a ranked list of mouse models for a chosen human disease (Chen et al. 2012; Hoehndorf et al. 2011; Smedley et al. 2013). Here we will describe the features available in some of the various tools developed by members of the Monarch Initiative before describing the Monarch Initiative website itself that integrates data from many other sources and allows users to visualize the phenotypic similarities.

### PhenoDigm

**Fig. 1:**
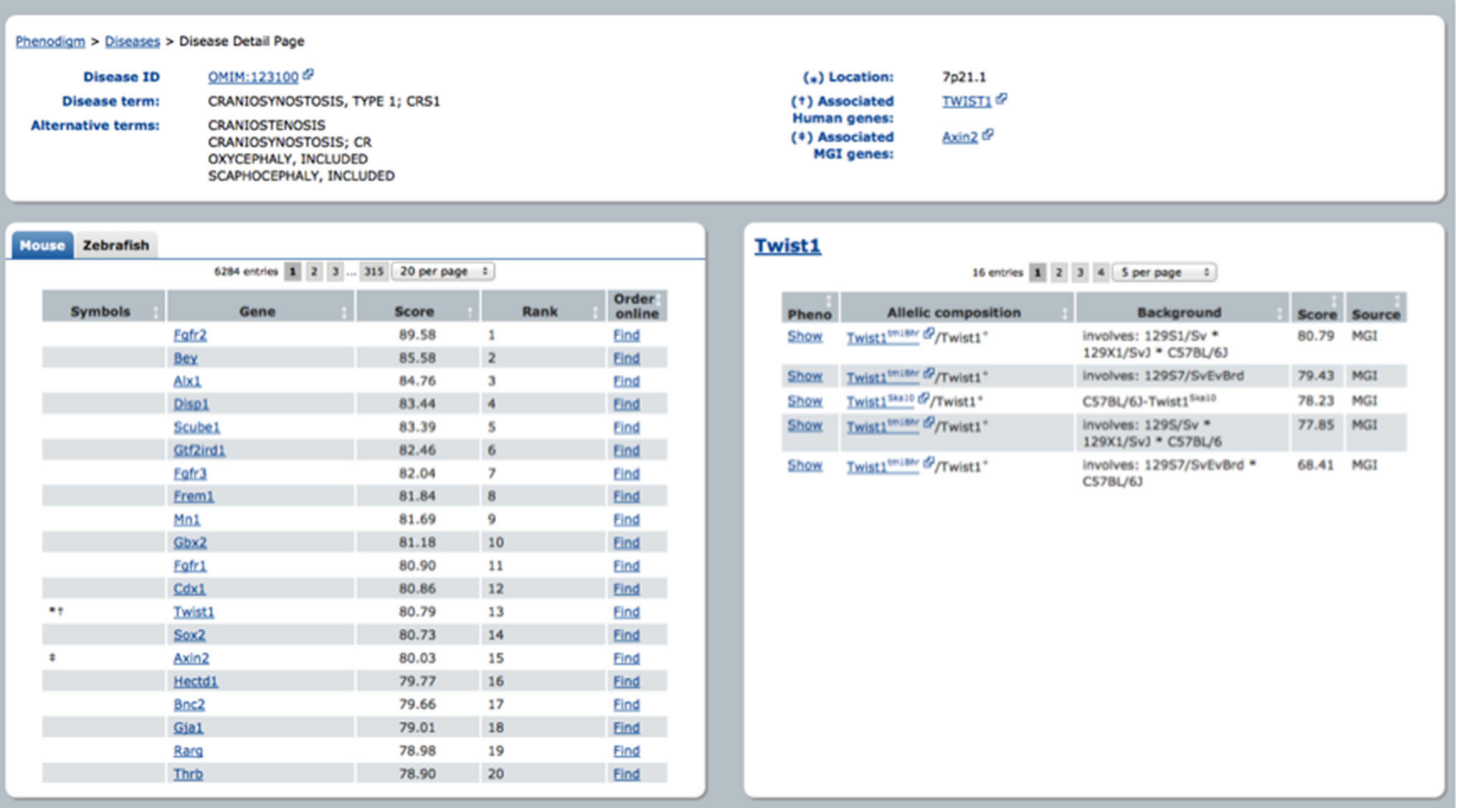
Cross-species phenotype comparisons using PhenoDigm identify an animal model for Craniosynostosis, type 1. Craniosynostosis, type 1 (OMIM:123100) is already known to be associated with mutations in *TWIST1* (*top panel*). The *bottom left panel* reveals that mouse mutants of *Twist1* represent a good phenotypic match to the clinical signs of this disease. The bottom right-hand panel shows the scores and evidence for different mouse mutants involving *Twist1*, allowing researchers to follow the *Order online* link to obtain the most relevant mouse strain for further mechanistic studies or therapeutic development

PhenoDigm allows users to query for copy number variant (CNV) syndromes from DECIPHER (Bragin et al. 2014) as well as rare diseases from OMIM and Orphanet. Ranked results from mouse and zebrafish phenotype comparisons are displayed along with the information on whether the mutation in the gene is known to be associated with the disease or is located in a critical region for diseases not yet associated with any gene. Clicking on a gene presents the results from individual animal models associated with that gene so the affect of different alleles, zygosity, and genetic background can be compared to select the optimal model. Many of these mouse models can then be ordered from public repositories for hypothesis-driven mechanistic or therapeutic target validation or purpose-driven therapeutic target effect experiments, e.g. the European Mouse Mutant Archive (Wilkinson et al. 2010). The individual matched phenotypes for each model can also be explored. Figure 1 shows an example where a disease (Craniosynostosis, type 1 OMIM:123100) associated with mutations of *TWIST1* is queried to discover that suitable *Twist1* mouse models of this disease exist and are available in public repositories. These tools can also be used to suggest candidate disease genes for diseases with no known molecular association.

### PhenogramViz

The cross-species phenotype comparison approach can also be used to assess the contribution of multiple genes within CNV regions to the disease phenotype (Doelken et al. 2013). Cases can be seen where the whole CNV syndrome can be explained by the disruption of only one of the affected genes, as well as others where different aspects of the syndrome are linked to different genes. PhenogramViz is a Cytoscape plug-in that allows clinicians to explore their own CNV patients by entering the deleted or duplicated region along with patient phenotypes (Köhler et al. 2014b).

### International Mouse Phenotyping Consortium

**Fig. 2:**
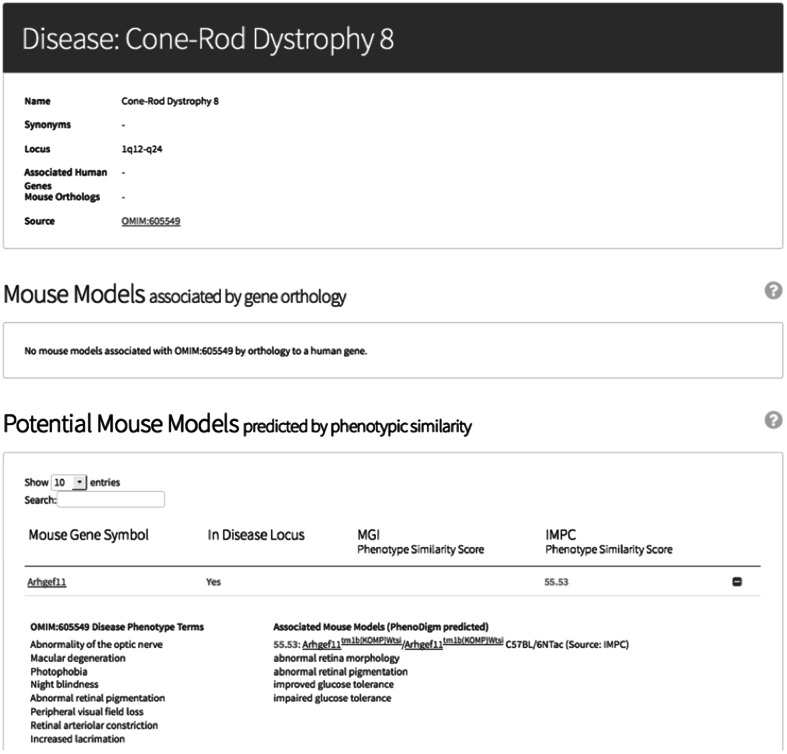
Identification of a novel candidate for Cone-Rod dystrophy 8 using cross-species phenotype comparisons at the IMPC portal. A high scoring phenotype match for OMIM:605549 is obtained for an IMPC mouse strain involving disruption of the mouse *Arhgef11* gene where abnormalities of the retina are reported in both the disease and the model. In addition, the tool highlights the human orthologue that lies within the previously reported locus at 1q12-24

Elsewhere in this issue, Meehan et al. describe the IMPC standardized phenotyping pipeline and portal. The data being generated by the IMPC’s controlled and robust statistical analysis framework are likely to be significantly more reproducible than literature-reported findings. Here, the IMPC has also mapped their quantitative assays to the MP, which enables semantic comparison using the PhenoDigm methodology to present high-quality, potential disease models in the IMPC pages. Rather than simple searches for results on individual diseases, faceted, combinatorial searches are allowed using factors such as disease category e.g. *cardiac*, and whether they are associated with known gene associations or with predicted associations from cross-species phenotype comparisons. Figure 2 shows an example where a novel candidate (*ARHGEF11*) is identified for Cone-Rod dystrophy 8 (OMIM:605549) based on phenotype matches to the IMPC model and the location of the gene in a previously identified critical region.

### The Monarch PhenoGrid

**Fig. 3:**
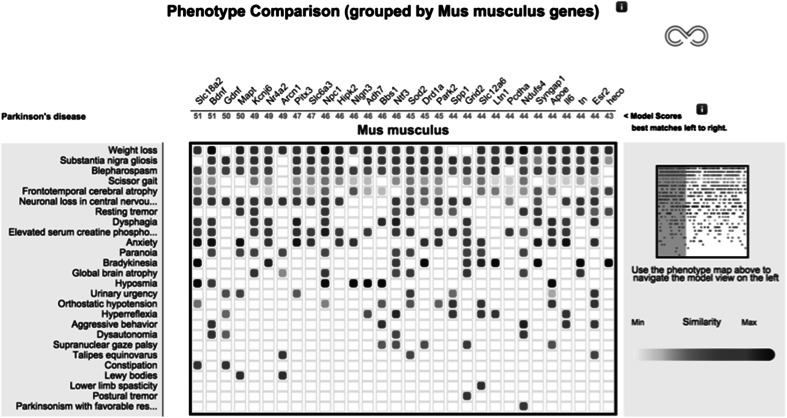
Monarch PhenoGrid showing a phenotypic comparison of Parkinson’s disease with the most phenotypically similar mouse models. Matching phenotypes are displayed in *rows*, matching models in *columns* (indicated here by the gene that is mutated), and *cell* contents *colour* coded with greater saturation indicating greater similarity. Mouse-over tooltips highlight diseases associated with a selected phenotype (or vice versa), or details (including similarity scores) of any match between a phenotype and a model. This example can be seen in the Compare tab at http://monarchinitiative.org/disease/DOID:14330 (Color figure online)

The integrated genotype–phenotype data held within Monarch can be utilized to drive the identification of models for disease research and disease diagnostics (as described above and for Exomiser below). Such integrated data can also be utilized for visualization of the relationships between the different data types. For example, PhenoGrid (Fig. 3), available on the Monarch website, highlights the phenotypic similarity of patient or disease profiles against the most similar mouse models. For software developers, PhenoGrid is available as an open-source widget suitable for integration in third-party websites (www.github.com/monarch-initiative/phenogrid), and is customizable with respect to organism, genotypes versus genes, and user-specified comparisons.

## Clinical application to rare disease diagnostics

Many incidences of rare disease remain undiagnosed after exome or genome sequencing due to the sheer number of candidate variants. Even after removing low quality and common variants and those deemed unlikely to be pathogenic, 10–100’s of variants remain. It is already known that each of us harbour ~100 genuine loss of function variants with ~20 genes completely inactivated (MacArthur et al. 2012), so prioritization based solely on variant frequency and pathogenicity is unlikely to identify the causative variant. The additional strategies of studying multiple-affected individuals, linkage data, identity-by-descent inference, de novo heterozygous mutations from trio analysis, or prior knowledge of affected pathways to narrow down to the causative variant are often not possible or successful.

In the last few years, a number of tools have been developed that utilize phenotype data associated with the patient as well as the results of sequencing (Javed et al. 2014; Robinson et al. 2014; Sifrim et al. 2013; Zemojtel et al. 2014). One of these tools, Exomiser, uses an algorithm termed PHenotypic Interpretation of Variants in Exomes (PHIVE) to combine data on the rarity of the variant and its predicted pathogenicity along with the similarity of the patient-to-mouse models for each candidate gene in the exome. A high scoring variant will be: (i) rarely or never observed in the 1000 Genomes Project and Exome Variant Server datasets, (ii) predicted to be highly pathogenic by PolyPhen, SIFT, and/or MutationTaster, and (iii) be located in a gene with a mouse model that exhibits very similar phenotypes to the patient.

For the phenotype comparisons, PHIVE uses the same OWLSim methodology used in the tools above and mouse phenotype data from MGI and IMPC. Benchmarking was performed on 100,000 simulated disease exomes containing known disease variants from HGMD added to unaffected exomes from the 1000 Genomes Project. The variant-based scores (frequency and pathogenicity) were found to combine synergistically with the phenotype scores to optimize the identification of the known causative variant as the top hit. The correct gene was recalled as the top hit in up to 83 % of samples and performance was improved by up to 54 fold by including phenotype information.

Although 88 % of the disease genes assessed had mouse strains with mutation in the orthologous gene, there were obviously some tested exomes where mouse phenotype data were missing and therefore performance will be expected to improve as the IMPC nears its goal of complete coverage of the genome. In the mean time, coverage has been increased by including human and zebrafish phenotypes as well as a guilt-by-association approach using protein–protein associations for those genes that have no data in any of the species. This modified algorithm (hiPHIVE) was able to detect the known disease-gene associations as the top hit in 97 % of the benchmarking exomes. In a strategy where the known human disease-gene phenotypes were masked, representing discovery of a novel association, the correct variant was detected as the top hit in 87 % of the benchmarking exomes. This version of Exomiser is being used by a number of groups as part of their analysis pipeline, such as the NIH Undiagnosed Disease Program (Gahl et al. 2012). The downloadable, command-line version of Exomiser requires no additional installation steps and is easily integrated into any bioinformatic pipeline.

## Conclusions

In this review we have highlighted the latest achievements in the computational analysis of mutations in mouse genes, mouse phenotypes, and mouse genotype–phenotype associations for novel insights into human disease. That any of this has been possible is testament to the remarkable ability of mouse models to recapitulate disease phenotypes, and the advances made in using ontologies to annotate and query disease and model organism data.

Improvements to the ontologies and algorithms are needed in particular disease areas (Oellrich et al. 2014; Robinson and Webber 2014). Beyond these technical challenges, a cultural shift is still needed to encourage collection of higher-quality phenotype data. For efficient and accurate diagnosis of rare disease patients, detailed and comprehensive clinical phenotypes need to be collected to be used alongside the new sequencing technologies in analysis (see http://monarch-initiative.blogspot.com/2015/01/how-to-annotate-patients-phenotypic.html for further detail). Use of tools such as PhenoTips (Girdea et al. 2013) can greatly facilitate informative patient phenotyping. On the mouse side, although IMPC will collect and annotate phenotype data on all protein-coding genes, the additional published phenotypes on these and other strains of mice will be vital for the successful interpretation of human genotype and phenotype data.

The role MGI plays in collecting these extra annotations will still be critical but the development of journal data submission rules for phenotypes would also be a welcome improvement. For example, if authors were required to describe all negative phenotypes (phenotypes measured but found to show no significant difference from wild type) then this highly relevant data could be incorporated into the phenotype matching algorithms. The Monarch Initiative is developing an online phenotyping tool to facilitate easy capture of phenotype data for any model organism and validate the genotypes with the correct nomenclature authorities. This will be critical to ensure publication of sufficient information to adequately link the phenotypic consequences of mutation to the specific genotype (Vasilevsky et al. 2013). The tool will also indicate whether or not the phenotypic profiles of the models are sufficient for comparison against all other known models of disease.

Assuming these challenges continue to be addressed, and with the completion of the IMPC’s dataset on functional consequences of mutation in all genes and the further development of these computational approaches, the next few years promise to be an exciting era for furthering our understanding of human disease by comparison analysis with mouse models.
